# Demonstration of a Three-dimensional Negative Index Medium Operated at Multiple-angle Incidences by Monolithic Metallic Hemispherical Shells

**DOI:** 10.1038/srep45549

**Published:** 2017-04-07

**Authors:** Ting-Tso Yeh, Tsung-Yu Huang, Takuo Tanaka, Ta-Jen Yen

**Affiliations:** 1 Department of Materials Science and Engineering, National Tsing Hua University, Hsinchu, Taiwan, ROC; 2 Department of Materials Science Center for Nanotechnology, Materials Science, and Microsystems, National Tsing Hua University, Hsinchu, Taiwan, ROC; 3 Metamaterials Laboratory, RIKEN, Saitama 351-0198, Japan; 4 RIKEN Center for Advanced Photonics, RIKEN, Saitama 351-0198, Japan; 5 Tokyo Institute of Technology, Kanagawa 226-8503, Japan

## Abstract

We design and construct a three-dimensional (3D) negative index medium (NIM) composed of gold hemispherical shells to supplant an integration of a split-ring resonator and a discrete plasmonic wire for both negative permeability and permittivity at THz gap. With the proposed highly symmetric gold hemispherical shells, the negative index is preserved at multiple incident angles ranging from 0° to 85° for both TE and TM waves, which is further evidenced by negative phase flows in animated field distributions and outweighs conventional fishnet structures with operating frequency shifts when varying incident angles. Finally, the fabrication of the gold hemispherical shells is facilitated via standard UV lithographic and isotropic wet etching processes and characterized by μ-FTIR. The measurement results agree the simulated ones very well.

The advent of negative index media (NIM)^1^,^2^ with non-existing material properties in nature has triggered a revolution in the field of electromagnetics and then enabled various intriguing applications such as perfect lenses^3^,^4^, negative Goos-Hänchen effect based slowing light systems^5^,^6^ and perfect absorbers^7^. The early demonstration of an NIM^1^,^2^ was realized via the combination of negative permeability from split-ring resonators (SRRs)^8^,^9^ and negative permittivity from plasmonic wires^10^. Yet, such combination is experimentally infeasible at a frequency higher than the microwave region and appears sensitive to the angle and polarization of the incident excitation. To ease the burden of fabrication and the limitation of polarization, a fishnet structure is introduced with intensive investigations by now^11^,^12^,^13^. The double negative identities of a fishnet structure, however, still suffer from the sensitivity of incident angles, for example, 8-degree off normal incidence alters the performance of the fishnet structure entirely^13^. Therefore, researchers are still eager to unravel such hindrance for an NIM operated under multiple incident angles beyond the microwave region. A rational solution is a three-dimensional (3D) NIM. There appear a few incumbent approaches to 3D NIMs, such as implementing six planar SRRs on six surfaces of a cube with orthogonally crossing continuous plasmonic wires embedded in a substrate^14^ and merging multi-directional V-shaped magnetic resonators and sphere-shaped electric resonators into a monolithic structure^15^. Nonetheless, both of the 3D NIMs confront the complexity of practical realization.

To resolve such circumscription, in this work we demonstrate a simple 3D NIM composed of an array of monolithic metallic hemispherical shells, which allows both negative permeability and negative permittivity under multiple-angle incidences within the THz gap. Besides, the employment of the metallic hemispherical shell guarantees a polarization independent response due to its circular symmetry under normal-incidence excitation. Most importantly, different from the antecedent 3D NIM^14^,^15^, such a metallic hemispherical shell array can be easily fabricated through standard UV lithographic and isotropic wet etching processes and will readily advance a variety of THz applications^16^,^17^.

## Simulation Methods and Experiment Procedure

To verify our design strategy, we employ a finite-integration solver of *CST Microwave Studio^TM^*, to calculate the complex scattering parameters of our proposed 3D NIM and then examine the retrieval results from the effective medium theory^18^, surface currents and phase propagation directions as well to affirm that our proposed structure possesses negative index under normal and oblique incidences.

**Figure 1:**
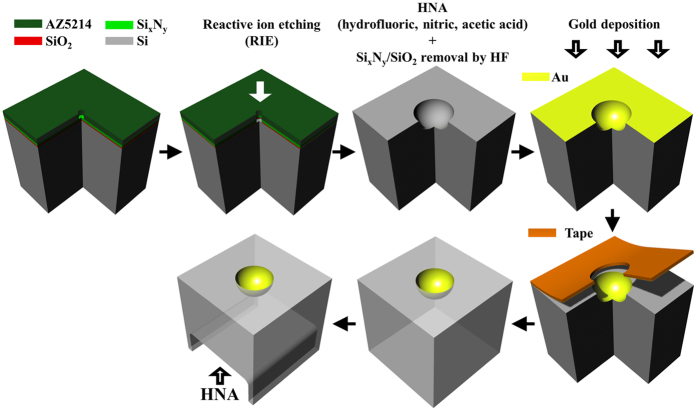
Schematic fabrication process of the gold hemispherical shell.

As for the fabrication of the 3D NIM, one can follow procedures as illustrated in Fig. 1. First, we spun a layer of 1.8-μm-thick photoresist (AZ5214) on a silicon substrate with silicon nitride/silicon oxide (500 nm/100 nm) as a mask to protect the substrate from etching, and a designed pattern, a circle with a 5-μm-wide diameter, is introduced onto the photoresist by standard UV lithographic process. Reactive ion etching (RIE) is utilized to remove silicon nitride/silicon oxide, and the sample was then dipped into an HNA (hydrofluoric acid, nitric acid, and acetic acid) solution with an etching rate of around 3.17 μm/min. After HNA etching, we can obtain a hemispherical cavity with a 34.17 ± 0.52-μm-wide diameter and then the residual silicon nitride/silicon oxide is removed by dipping the sample into 49 wt% hydrofluoric acid. In the following, we deposited a continuous 100-nm-thick gold film by an electron beam evaporator. Note that before depositing a gold film, the sample is dipped into 69 wt% nitric acid for 4 hours in order to form a thin layer of silicon oxide, which can benefit the following peeling off process. Afterward, we stuck a tape on the top surface of the sample and peeled off the gold film on the surface but left gold well-attached to the hemispherical cavity; finally, we utilized HNA to etch the back of the supporting silicon substrate down to 50-μm, thus completing the fabrication process of the gold hemispherical shells.

Finally, we utilized a micro Fourier transform infrared spectrometer (Bruker Vertex 70 V μ-FTIR) to measure the relative transmission of the fabricated samples at 0°, 15° and 30° incidences by our homemade stage. These samples were illuminated by a mercury lamp through an aperture of 8 mm in diameter, and a PE/DTGS detector was placed on the opposite side to collect the transmitted signals. All measurements were done in vacuum to reduce the noise from the ambient environment including absorption by moisture and air.

## Results and Discussion

### Structure design of 3D NIM and its S-parameters

**Figure 2:**
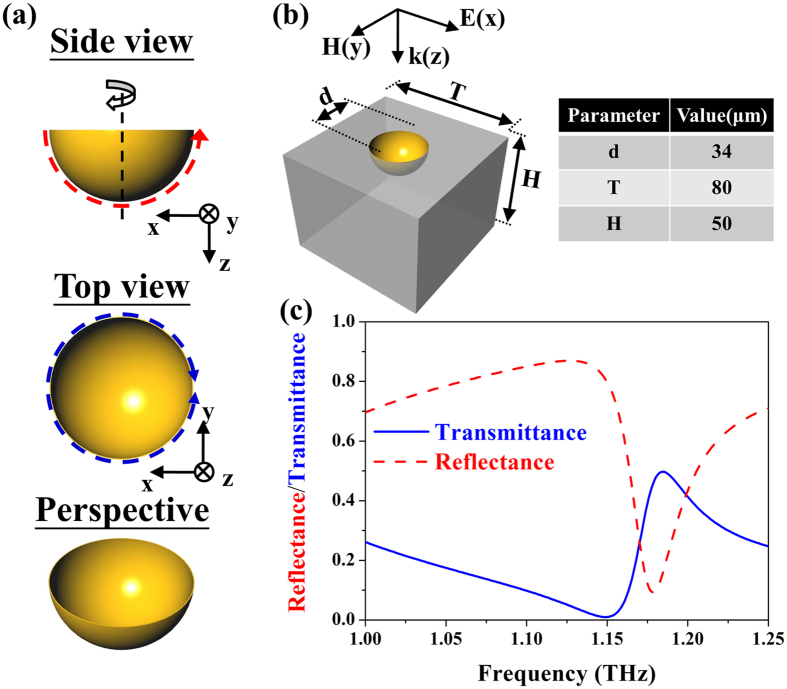
A unit cell of three-dimensional negative index media and its S-parameters. (**a**) Top, side and perspective views of a gold hemispherical shell; red dashed line indicates the constructing component, i.e., a C-shaped split-ring resonator. Further, the projective area on x-y plane of the shell could function as a metallic closed ring denoted by blue dashed circle. (**b**) Designed gold hemispherical shell and its dimensional parameters. The metallic semispherical shell is made of a 100-nm-thick gold (yellow part) with conductivity of 4.56 × 10^7^ S/m and embedded in a 50-μm-thick lossy silicon substrate (grey part) with permittivity of 12 and conductivity of 8 S/m. (**c**) Simulated scattering parameters of the shell.

Our proposed 3D NIM, the gold hemispherical shell is presented in Fig. 2(a), which is developed from rotating a C-shaped SRR denoted by a red dash arrow along the symmetry-axis 180-degree. Therefore, this gold hemispherical shell is capable of furnishing negative permeability under arbitrary polarization according to its circular symmetry. In addition to negative permeability, the gold hemispherical shell also possesses negative permittivity even under high incident-angle cases since the projective area denoted by the blue dash circle of the gold hemispherical shell can be intuitively regarded as a metallic closed ring^19^,^20^ for each incident angle. Consequently, the monolithic gold hemispherical shell solely can supplant the assembling of conventional SRRs and discrete plasmonic wires to maintain double negative identities under various incident angles. Herein, our designed gold hemispherical shell is made of a diameter of 34 μm, periodicity of 80 μm and a 100-nm-thick gold layer, deposited on a silicon substrate with a thickness of 50 μm as detailed in the table of Fig. 2(b). Such structure is within the fabrication capability of UV lithographic and isotropic wet etching processes. To our best knowledge, it is the very first work to demonstrate an NIM from 0° up to 85° incident angles in numerical simulation and in experimental measurement.

To validate and to characterize our design, we employed a *CST Microwave Studio^TM^*, to calculate the complex scattering parameters. For the metallic hemispherical shell, we chose an excellent conductor of gold with conductivity (σ) of 4.56×10^7^ S/m due to its lower losses in the THz region and inert chemical properties beyond copper and silver, respectively; for the supporting substrate, we adopted a 50-μm-thick silicon (ε_r_ = 12 and σ = 8 S/m)^21^ that is suitable to the isotropic wet etching process. First of all, the performance under the normal incidence is introduced in Fig. 2(c), in which the electromagnetic wave vector (k) is parallel to the z-direction and the external E-field is lying on the x-y plane (indicated in Fig. 2(b)). Figure 2(c) presents the simulated transmittance and reflectance spectra, indicated by blue solid and red dot lines, respectively. There appears a transmittance dip at the frequency of 1.149 THz, gradually elevating to a transmittance apex at 1.185 THz, where we expect to observe double negative identities^13^,^14^,^15^.

### Characterization of the 3D NIM under normal incidence

**Figure 3:**
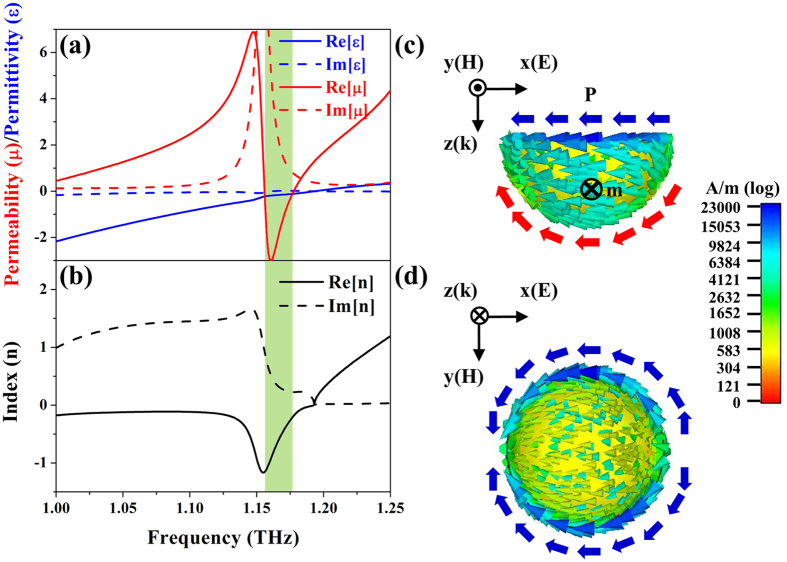
Retrieval results and surface current distributions of the shell. (**a**) Retrieved permittivity, permeability and (**b**) index of the gold hemispherical shell. (**c**) Side-view (x-z plane) and (**d**) top-view (x-y plane) of surface currents on the shell. A current loop at the bottom in (**c**) suggests a negative magnetic dipole, **m**; meanwhile, surface currents along the edge of the shell in (**d**) induce a negative electric dipole, **P**.

To certify our prediction, the corresponding constitutive parameters are retrieved to verify the existence of the double negative identities, i.e., a negative index^18^ as shown in Fig. 3(a,b). Figure 3(a) reveals a Drude-model-like behavior of an electric resonance, i.e., negative permittivity before 1.193 THz and anti-resonance at 1.149 THz due to finite spatial periodicity^22^; in contrast, the occurrence of magnetic resonances forms a Lorentz model resonance and reveals negative permeability between the frequencies of 1.156 THz and 1.177 THz. These double negative identities result in a negative index at the similar frequency regime as highlighted by a green area in Fig. 3(b) which overlaps with the frequency range of the transmission maximum where both permittivity and permeability are negative. The coincidence of the negative index band and the transmission maximum confirms our abovementioned expectation^23^ and herein we affirm that a transmission apex gradually elevating from a dip is our indicator for a negative index.

To further interpret the magnetic and electric resonances in the monolithic gold hemispherical shell, we scrutinize the distributions of surface currents at the resonance frequency (i.e., 1.149 THz) as illustrated in Fig. 3(c,d). At 1.149 THz, the side-view (x-z plane) of the surface currents portrayed in Fig. 3(c) displays a current loop along the circumference of the shell to form a negative magnetic dipole with respect to incident waves that provides negative permeability as suggested by the retrieval results. On the other hand, according to side- and top-views of the current distributions (x-y plane) in Fig. 3(d), there appears oscillating surface currents along the edge of the shell, generating a negative electric dipole and offering negative permittivity.

**Figure 4:**
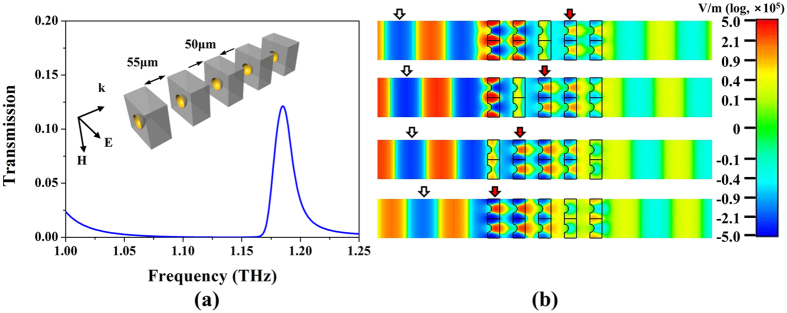
Phase flows within free space and five-layered 3D NIM under normal incidence. (**a**) Simulated S-parameter of five-layered gold hemispherical shells with 55-μm separation. (**b**) Illustration of phase propagation at 1.185 THz with a constant step of phase, i.e., 50-degree, from the upper to the lower frames. The direction of the phase flow within the gold hemispherical shells (denoted by red arrows) is opposite to the direction of incident waves in the free space (denoted by white arrows), an indication of negative index.

Finally, we monitor the animated field distribution of the gold hemispherical shells to identify the negative index by revealing the opposite propagation direction of the phase flows across the NIM and free space^24^,^25^. To clearly observe the fields, we stack five-layer gold hemispherical shells with a 55-μm separation and compare the directions of the phase flow within the gold hemispherical shells to free space. After assembling such five-layered 3D NIM, the value of the transmission apex at around 1.185 THz becomes smaller as shown in Fig. 4(a) compared to the one with the single-layered 3D NIM because of much higher losses from the five-layered one. The animated phases of electric field at 1.185 THz are portrayed in Fig. 4(b), and the direction of the phase flow within the five-layered NIM is indeed opposite to the one in free space if we just follow the red arrows in Fig. 4(b) that move from right to left with a constant step of 50-degree phase change in the sequential upper to lower frames and whose movement is opposite to white arrows, i.e., the corresponding phase flow in free space. The illustration of the negative phase flow confirms the existence of the negative index for our proposed gold hemispherical shell array under the normal incident condition.

### Characterization of 3D NIM under oblique incidence

**Figure 5:**
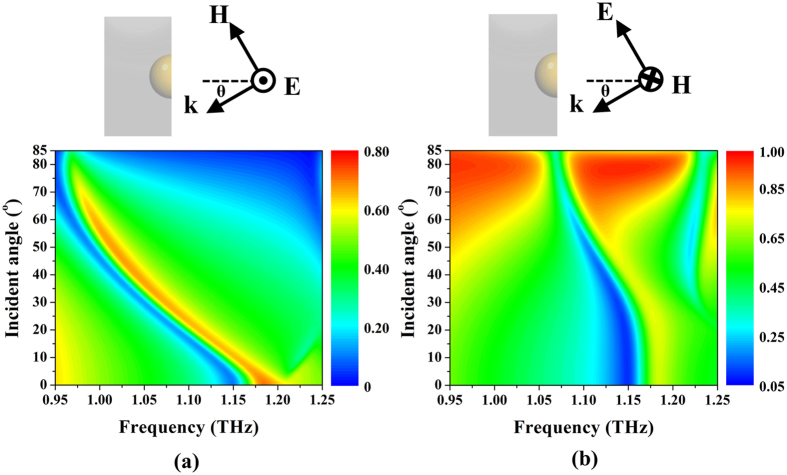
Transmission spectra of the single-layered 3D NIM under oblique incidence. Simulated transmission contour with respect to frequency and incident angle of a single-layered 3D NIM for (**a**) TE and (**b**) TM cases, respectively. Insets define configurations of TE and TM cases.

The success in developing the 3D NIM under the normal incident condition by the gold hemispherical shell array encourages us to further examine the shell under oblique incident conditions. In the following, we would elaborate transmission apexes of the single-layered 3D NIM to identify the double negative identities under oblique incidence from 0° up to 85° for both TE and TM cases (see Fig. 5(a,b) for the definition of the TE and the TM cases, respectively). For the TE case, a transmission apex with double negative identities maintains its amplitude and shifts toward to the lower frequency with a frequency offset of 0.0028 THz/degree. Such a red shift corresponds to a lower resonance frequency of magnetic dipoles, stemming from a longer surface current loop at the bottom^26^,^27^. On the contrary, for the electric resonance, the surface current distribution on the edge of the shell under oblique incidence remains similar so that the resonant frequency of the electric resonance is unaltered (see Fig. S1 in Supporting Information). On the other hand, for the TM case, the transmission apex preserves its amplitude as the same as the TE case but only with a slightly red-shifted operating frequency around 0.0001 THz/degree within the first 20° oblique incidence (linear region) that is much smaller compared to the TE case and indicates the double negative identities from our shell is less sensitive to the incident angle in the TM case than in the TE case. Such incident-angle insensitivity in the TM case stems from a combination of an imperceptibly lengthened current loop and a similar electric dipole profile compared to the normal incident ones when increasing incident angles (see Fig. S1). Overall, our proposed monolithic 3D NIM, composed of monolithic gold hemispherical shells, is superior to the NIM from the combination of SRRs and plasmonic wires^1^,^2^ and fishnet structure^13^,^28^ for its less sensitivity to polarization and incident angles, respectively.

**Figure 6:**
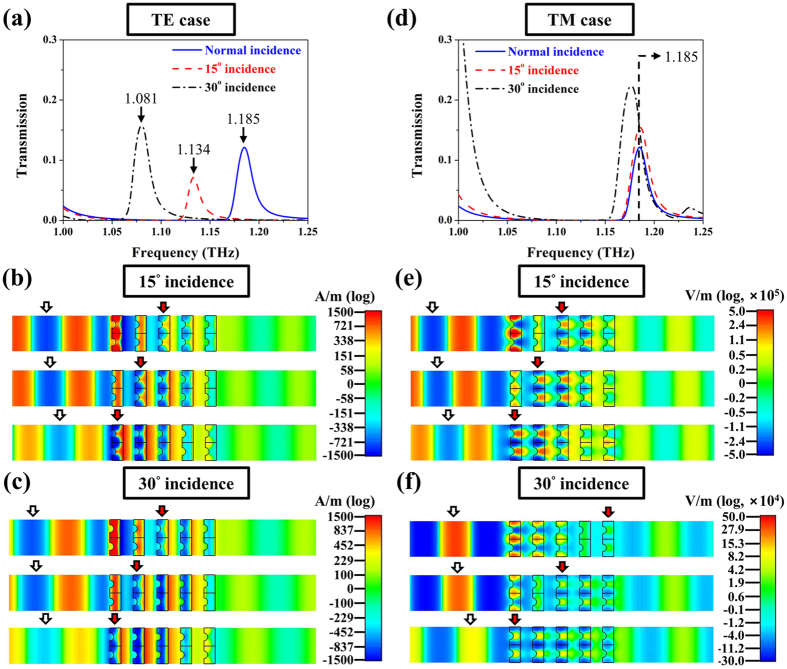
Phase flows within free space and five-layered 3D NIM under oblique incidence. Simulated transmission spectra of five-layered 3D NIMs under 0°, 15° and 30° incidences for (**a**) TE and (**d**) TM cases. The animated phases of magnetic fields under (**b**) 15° incidence at 1.134 THz and (**c**) 30° incidence at 1.081 THz and the animated phases of electric fields under (**e**) 15° incidence and (**f**) 30° incidence both at 1.185 THz are presented. In order to clarify the wave propagating direction, we monitor different fields, magnetic fields for TE case and electric fields for TM case so that the phase of fields could be plane-wave-like. The upper to lower frames in (**b**,**c**) to (**e**,**f**) are all with a constant step of phase change, i.e., 50-degree, and white arrows indicate the wave propagation direction in free space while red arrows in five-layered 3D NIM. The two are opposite and suggest the double negative identities of the 3D NIM.

Next, we record the phase flow of within the 3D NIM under the oblique incident cases and exemplify the negative index at 15° and 30° incident angles for both TE and TM cases. The transmission spectra and animated phases of the five-layered 3D NIM at 15° and 30° incidences are portrayed in Fig. 6(a–c) for the TE case, and Fig. 6(d–f) for the TM cases. The simulated transmission spectra (see Fig. 6(a,d)) of the five-layered 3D NIM display a red shift of the transmission peaks for the TE case but stay almost unaltered for the TM case from normal to 30° incidence that is consistent with the trend of the single-layered 3D NIM. In the following phase flow demonstration, we monitor different fields, magnetic fields for the TE case and electric fields for the TM case so that phases of the fields could be plane-wave-like to be clearly observed. Figure 6(b,c) show the snapshots of the animated phases for the transmission apexes at 1.134 THz and at 1.081 THz at 15° and 30° incidences, respectively for the TE case; Fig. 6(e,f) display the flows at the frequency of 1.185 THz under the two incidences for the TM case. Within all the consecutive snapshots of the animated phases from the upper to lower frames, the wave propagation directions in free space (white arrows) and in the five-layered 3D NIM (red arrows) evidently reveal opposite phase flows to corroborate the negative index.

Note that this 3D NIM possesses a negative index at least from 0° to 85° incidences for both TE and TM cases (see the phase flows under 60° and 85° incidences in Fig. S2). Especially for the TM case, negative phase flows can exist almost at the same frequency (i.e., 1.185 THz) up to 85° incidence. In summary, these simulation results indicate that the proposed 3D NIM maintains a negative index from normal (i.e. 0° incidence) up to 85° incidence regardless of the TE or the TM cases.

### Fabrication and measurement results

**Figure 7:**
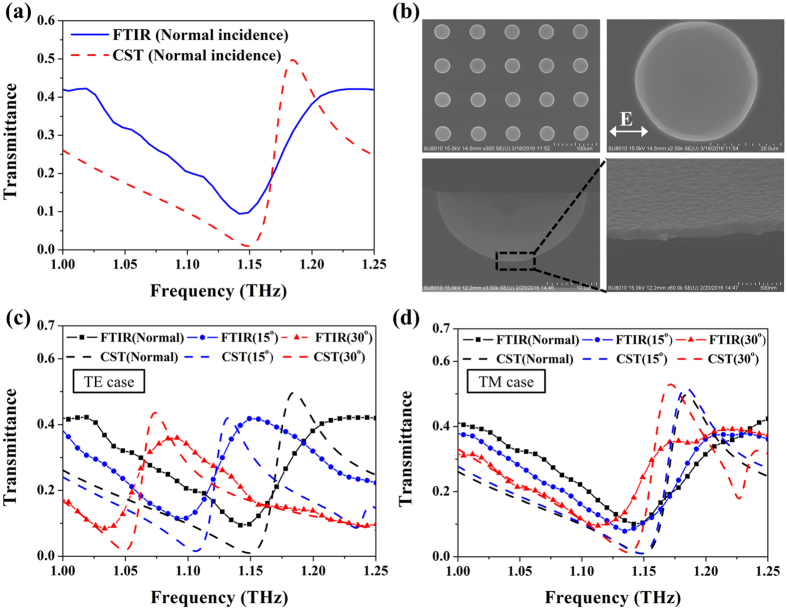
Measured S-parameters and SEM images of the fabricated 3D NIM. (**a**) Transmittance spectra between μ-FTIR measurement and numerical simulation under normal incidence, (**b**) scanning electron microscope images of the fabricated gold hemispherical shell indicating the distribution of diameter in shell is approximately 34.17 ± 0.52 μm and μ-FTIR measurement results of the gold hemispherical shell for (**c**) TE case and (**d**) TM case at 0°, 15° and 30° incidence.

To materialize the designed 3D NIM made of the gold hemispherical shell, we employed standard UV lithographic and isotropic wet etching processes. The fabricated sample is characterized by micro-Fourier transform infrared microscopy (μ-FTIR) with a homemade platform to control various incident angles. The measured transmittance spectrum of the gold hemispherical shell under normal incidence is shown in Fig. 7(a). There exists a transmittance dip at 1.142 THz, and an apex at 1.229 THz to identify the negative index. The measurement agrees well with the simulation, with a little offset in terms of frequency location (5.2% for the apex location) and resonance efficiency (15.2% for the apex transmittance). Such an offset is mainly from the fabrication imperfection of the size and the shape of the shells and the surface roughness of the deposited gold as shown in Fig. 7(b), which could be fitted by changing the parameters such as thickness of Si-substrate, conductivity of Si and the damping frequency of metal (See Fig. S4 in Supplementary Information). Although there exist absorption losses in the gold shell, the performance of the 3D NIM is still preserved as presented in Fig. 7(c,d). In Fig. 7(c) for the TE case, a red shift of the transmission peak with an offset of 0.0048 THz/degree is observed when increasing the incident angle; on the other hand, in Fig. 7(d) for TM case, the transmission dip is slightly red-shifted with the offset of 0.0017 THz/degree from normal to 15° incidences. Based on the trend of the transmission dip, the μ-FTIR measurement results agree well with the simulation, and the transmission dip in the TM case is more insensitive to the incident angle compared to the one in the TE case. Therefore, the 3D NIM, i.e., the gold hemispherical shells, operated at multiple angle incidences, i.e. 0°, 15° and 30° is experimentally demonstrated.

## Conclusion

We demonstrated an NIM by the monolithic gold hemispherical shells in both numerical simulation and experimental verification. Such monolithic gold hemispherical shells are the three-dimensional superposition of conventional SRRs for negative permeability and discrete plasmonic wires for negative permittivity, allowing a negative index operated at multiple-angle incidences. Due to the circular symmetry of the hemispherical shell, this deliberately designed NIM is insensitive to the polarization of incident light; meanwhile, the 3D NIM maintains its negative index under the oblique incident angles spanning from 0° to 85° for both TE and TM cases, evidenced by the negative phase flows within the five-layered shells. Our designed monolithic gold hemispherical shells are fabricated via standard UV lithographic and isotropic wet etching processes, and then measured by an evacuated μ-FTIR system. Both simulation and experiment were in a good agreement. To our best knowledge, this is the very first work to experimentally demonstrate a feasible 3D NIM at THz ranges. Such an NIM comprised of monolithic gold hemispherical shells can ease the burden of the strong anisotropic responses in the conventional planar metamaterials and widens the route to practical applications including superlenses, slowing light by negative Goos-Hänchen effect and perfect absorbers.

## Additional Information

**How to cite this article**: Yeh, T.-T. *et al*. Demonstration of a Three-dimensional Negative Index Medium Operated at Multiple-angle Incidences by Monolithic Metallic Hemispherical Shells. *Sci. Rep.* **7**, 45549; doi: 10.1038/srep45549 (2017).

**Publisher's note:** Springer Nature remains neutral with regard to jurisdictional claims in published maps and institutional affiliations.

## Supplementary Material

Supplementary Information
