# Supplementary material for: Demonstration of a Three-dimensional Negative Index Medium Operated at Multiple-angle Incidences by Monolithic Metallic Hemispherical Shells
Source: Sci Rep. 2017 Apr 7;7:45549. doi: 10.1038/srep45549 (PMC5384282; doi:10.1038/srep45549)
Supplement: Supplementary Information [file srep45549-s1.pdf]

Demonstration of a Three-dimensional Negative Index Medium Operated at Multiple-angle Incidences by Monolithic Metallic Hemispherical Shells

(Running title: A Negative Index Medium Operated under Oblique Incidence)

Ting-Tso Yeh[1], Tsung-Yu Huang[1] and Ta-Jen Yen[1,2]

[1]Department of Material Science and Engineering, National Tsing Hua University, Hsinchu, Taiwan, R.O.C.

[2]Department of Materials Science Center for Nanotechnology, Materials Science, and Microsystems, National Tsing Hua University, Hsinchu, Taiwan, R.O.C.

Correspondence: *Prof. Ta-Jen Yen, Department of Materials Science and Engineering, National Tsing Hua University101, Section 2, Kuang Fu Road, Hsinchu 30013, Taiwan, R.O.C.

Email: tjyen@mx.nthu.edu.tw

Keywords: multiple-angle incidences; negative index; terahertz; three-dimensional metamaterials

- **Mechanisms of red-shifted resonant frequency at normal, 15°and 30° incidences based on surface current distributions of the gold hemispherical shell**

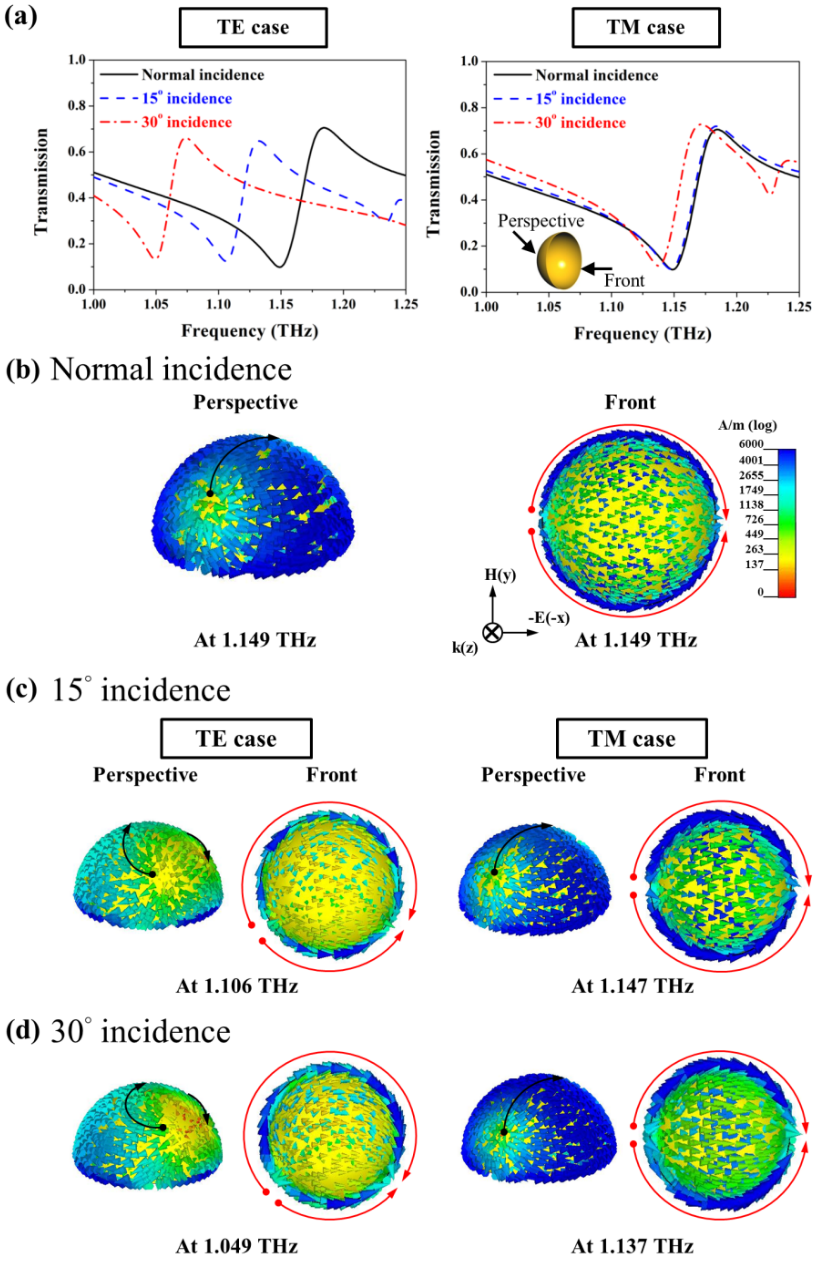

Figure S1. (a) Transmission spectra of the gold hemispherical shell at normal, 15° and 30° incidences for the TE and TM cases (black for normal incidence, blue for 15° incidence and red for 30° incidence, respectively). Inset shows the corresponding perspective- and front-views displayed in (b)-(d). Surface current distributions for perspective-view (left) and front-view (right) of the gold hemispherical shell at (b) normal, (c) 15° and (d) 30° incidences for the TE and TM cases at their corresponding resonant dip. Note that, (b)-(d) share the same scale bar.

To explain mechanisms of the red-shifted resonant frequency for both TE and TM waves when increasing incident angles, we monitor surface current distributions of the shell at normal, 15° and 30° incidences at the corresponding resonant dips (see Figure S1(a)) as shown in Figure S1(b)-(d). As plotted in Figure S1(b), for normal incidence, the currents at the bottom center denoted by a black arrow majorly contribute to the magnetic dipoles along y direction. Furthermore, induced surface currents along the edge of the shell denoted by red arrows lead to electric dipoles as mentioned in the main text. Therefore, our proposed shell could possess double negative identities at 1.149 THz from the combination of the induced magnetic and electric dipoles. Now, we would like to discuss cases for oblique incidences. First of all, for the TE case (left column from Figure S1(c)-(d)), when the incident angle tilts from 0° to 15°, the surface current distributions become asymmetrically distorted and form a greater length of the loop of the induced currents denoted by a black arrow in Figure S1(c) than the case under normal incidence so that there still exist magnetic dipoles, yet resonating at a lower frequency. Even the induced currents at the edge become asymmetry compared to the normal incident case, the net electric dipoles are still along x-direction, resulting in a small shift of the plasma frequency of Drude-model-like resonance and possessing insignificant influences on the regime of the negative index. Combining the frequency shifts of the magnetic and electric dipoles, the region of the negative index shifts to a lower frequency, i.e., 1.106 THz when the incident angle is up to 15°. To further increase the incident angle to 30°, a much larger loop denoted by black arrows as illustrated in Figure S1d and similar electric dipoles give rise to red-shifted negative index accompanied with a red-shifted resonant dip at 1.049 THz. As for the TM case (right column in Figure S1(c)-(d)), the surface current distributions at 15° and 30° incidences are both similar to the distribution in the normal incidence case but with a little longer path at 15° and 30° incidences as suggested by the closer distance between the node and the edge at 30° incidence. Consequently, the

magnetic resonance barely red-shifts to 1.147 THz at 15° incidence and to 1.137 THz at 30° incidence compared to 1.149 THz under normal incidence. On the other hand, the current distributions that form electric dipoles at both 15° and 30° incidences are the same as the distributions under normal incidence; thus, the electric resonance fixed at the same frequency under the oblique incidences. Therefore, the slightly red-shifted resonant dip for TM case under oblique incidences is caused by imperceptibly longer paths of the induced surface currents, thus resulting in a magnetic dipole resonance at a lower frequency and similar current profiles on the edge of the shell, leading to an electric dipole similar to the normal incident one when increasing the incident angle.

- ## The phase flows of the five-layered 3D NIM at 60° and 85° incidence

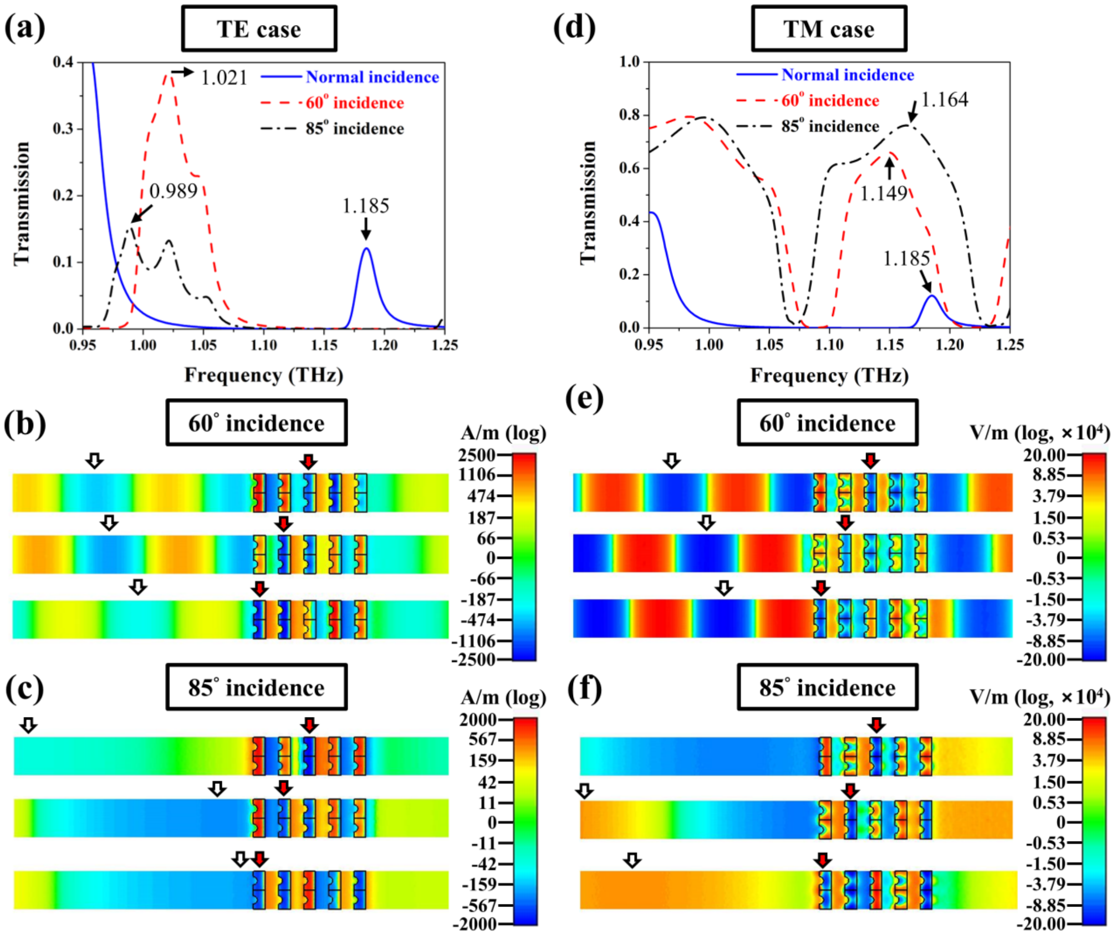

Figure S2. Simulated transmission spectra of five-layered 3D NIMs at normal, 60° and 85° incidences for the (a) TE and (d) TM cases, respectively. The animated phases of magnetic fields under (b) 60° incidence at 1.021 THz and (c) 85° incidence at 0.989 THz for the TE case, and the animated phases of electric fields under (e) 60° incidence at 1.149 THz and (f) 85° incidence at 1.164 THz for the TM case. The upper to lower frames in (b)-(c) and (e)-(f) are all with a constant step of phase change, i.e., 70-degree, and white arrows indicate the wave propagation direction in free space while red arrows in five-layered 3D NIM. The two are opposite and suggest the double negative identities of the 3D NIM.

The transmission spectra and animated phases of the five-layered 3D NIM at 60° and 85° incidences are portrayed in Figure S2(a)-(c) and S2(d)-(f) for the TE and the TM cases, respectively. The simulated transmission spectra (see Figure S2(a) and S2(d)) of the five-layered NIM display distinguishable apexes where we could monitor their animated phase.

Figure 6(b) and 6(c) show the snapshots of the animated phases at the transmission apexes of 1.021 THz and 0.989 THz under 60° and 85° incidences, respectively for the TE case; Figure 6(e) and 6(f) display the flows at the frequencies of 1.149 THz under 60° incidence and of 1.164 THz under 85° incidence, respectively for the TM case. Within all the snapshots of the animated phases, the wave propagation direction in free space is denoted by white arrows; in contrast, the wave propagation direction within the five-layered 3D NIM is denoted by red arrows and from the upper to lower frames, the white arrows go from left to right when the red arrows go from right to left, an indication of opposite phase flows and negative index as well. Herein, our proposed 3D NIM, the gold hemispherical shell, theoretically preserved double negative identities up to 85°.

● Performance of our metallic shells incident from two opposite sides

Once the sample is flipped, we could still obtain identical transmission and similar reflection compared to the one with the light shedding from the front side under normal and oblique incidences as shown in Fig. S3.

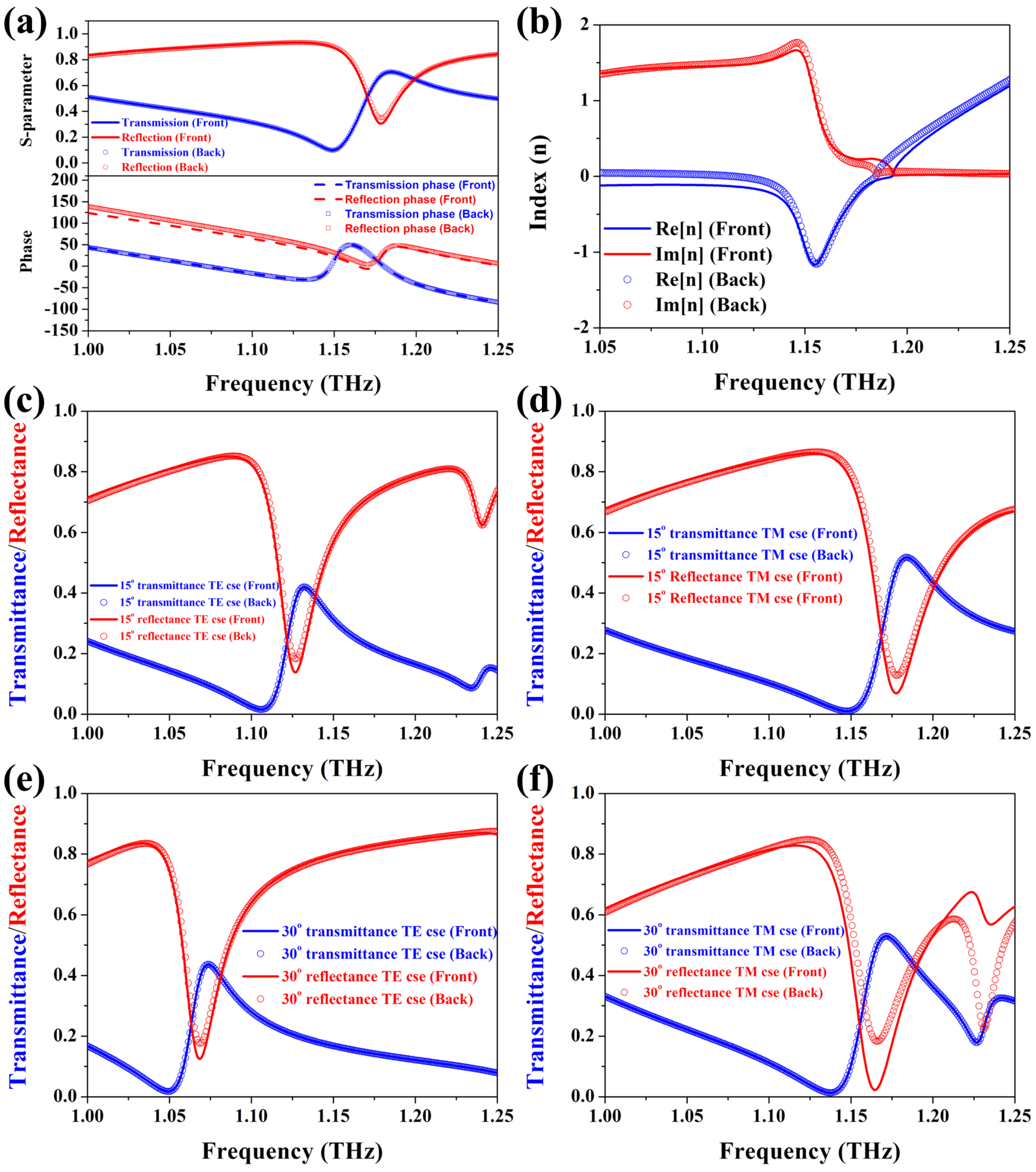

Figure S3. (a) Simulated transmittance and reflectance spectra of the gold shells under normal incidence and (b) their retrieval results from front-side and back-side. Transmittance and Reflectance spectra under (c) 15° incidence, (d) 30° incidence in the TE case and (d) 15° incidence and (e) 30° incidence in the TM case from front-side and back-side.

● Revised parameters in simulation for a better fit to the experimental results

Our experimental demonstration agrees well the numerical simulation with only a little offset (i.e., 5.2% for the frequency offset and 15.2% for the transmission offset). The offset stems from the deviated thickness of Si substrate caused by Si back etching, and the roughness of gold that is impractical to estimate before the experiments. We can modify our simulation parameters to further approach the experimental results by changing the thickness of Si substrates, damping frequency of metal and the conductivity of the Si substrate as shown in Fig. S4.

(a)

	Modified	Original
Thickness of Si (μm)	46	50
Conductivity of Si (S/m)	2	8
Damping frequency of gold ($\times 10^{12}$ Hz)	2002.05	-

(b)

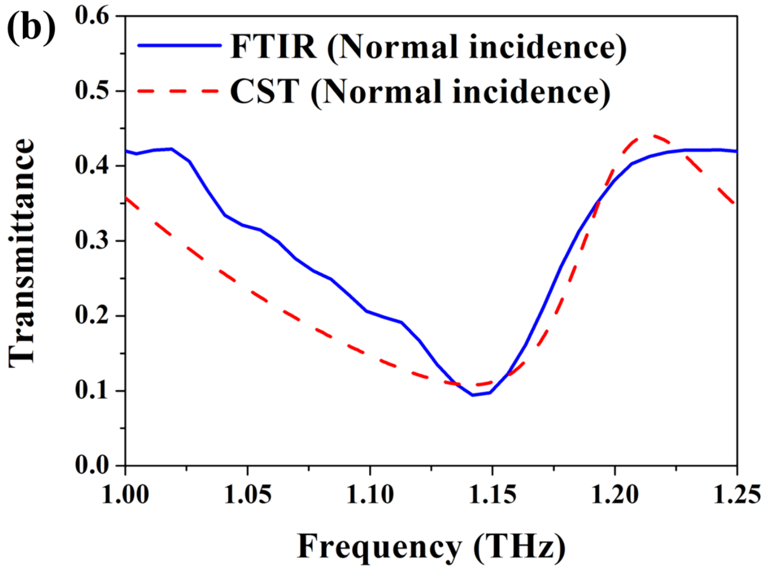

Figure S4. (a) Modified and original parameters of 3D metamaterials including the thickness and conductivity of the silicon substrate and the damping frequency of gold. Note that the modified damping frequency of gold is set 450-times greater than the value provided in the [R3]. (b) Comparison between simulated and measured transmittance spectra based on the modified parameters.

- **Misalignment when piling up to five-layered 3D metamaterials**

Limited by our measurement facilities, we can only measure a single-layered sample under 0° (i.e., normal), 15° and 30° incidences for both TE and TM waves, and then compared them to the simulated ones, as shown in Fig. 7 in the main text. Again, the results of simulation and measurement agree one another well, demonstrating negative refraction at multiple-angle incidences by our designed monolithic metallic hemispherical shells. Besides, single-layered metallic hemispherical shells suffice to be regarded as a negative refractive index medium (NRIM). The reason that we employed a five-layered configuration instead of a single-layered configuration is to visualize the opposite phase flows in the structures and in free space to indicate negative refraction. Therefore, the measurement of a five-layered sample is not a must, because one cannot directly observe the phase flow in structures.

Furthermore, we can also predict the behavior of the five-layered sample under misalignment by simulation, as shown in Fig. S5 and S6. We simulated the cases of different lateral shifts in x-direction only and in both x- and y-directions and of tilted angles (two layers with 15° tilted angle). As shown in Fig. S5, the lateral shifts have no significant impact on the transmittance spectrum. In addition, the tilted angles would introduce a small frequency shift but overall transmittance looks similar to the case of the well-aligned one as shown in Fig. S6. From both Fig. S5 and S6, we believe that our design is not sensitive to the misalignment supposed one plans to pile-stack more layers of 3D metamaterials for practical applications.

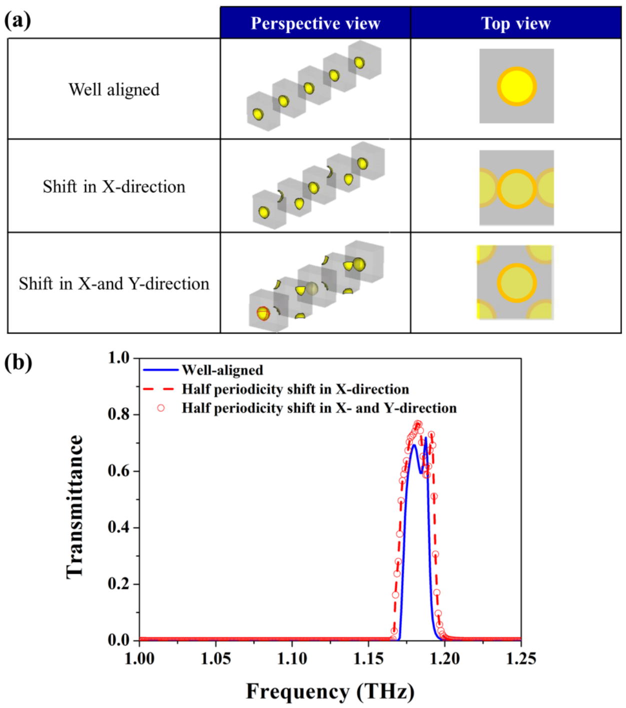

Figure S5. (a) Schemes of shifted 3D metamaterials in x-direction only and in both x- and y-direction when piling up to 5-layered bulk ones. (b) Corresponding transmittance spectra suggest a minor offset compared to the well-aligned one. We believe that lateral shifts would not destroy the negative index provided by our 3D metamaterial.

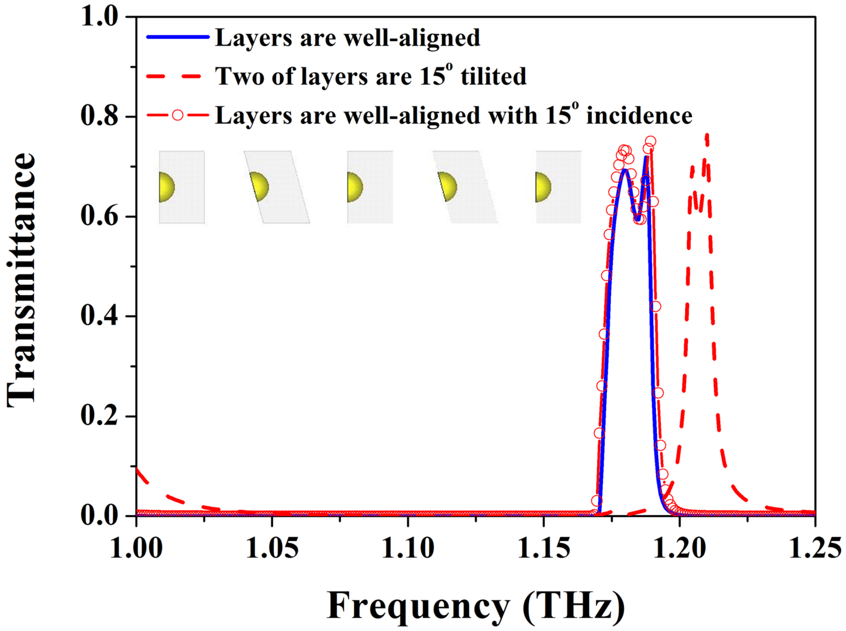

Figure S6. Transmittance spectra of our five-layered metamaterial with well-aligned layers and two 15°-tilted layers among the five. The two shares similar transmittance profile with only a frequency shift; thus, we believe that the behavior of negative index is preserved even for two 15°-tilted layers.
